# One Plus One Equals Two—will that do? A trial protocol for a Swedish multicentre randomised controlled trial to evaluate a clinical practice to reduce severe perineal trauma {1}

**DOI:** 10.1186/s13063-020-04837-7

**Published:** 2020-11-23

**Authors:** M. Edqvist, H. G. Dahlen, C. Häggsgård, H. Tern, K. Ängeby, G. Tegerstedt, P. Teleman, G. Ajne, C. Rubertsson

**Affiliations:** 1 Department of Health Sciences, Faculty of Medicine, Lund University, Lund, Sweden; 2 Clinical Epidemiology Unit, Department of Medicine, Karolinska Institutet, Stockholm, Sweden; 3 School of Nursing and Midwifery, Western Sydney University, Sydney, Australia; 4 Centre for Clinical Research and Education, Region Värmland, Karlstad, Sweden; 5 School of Education, Health and Social Studies, Dalarna University, Karlstad, Sweden; 6 Department of Obstetrics and Gynaecology, CLINTEC, Karolinska Institutet, Stockholm, Sweden; 7 Department of Obstetrics and Gynaecology, Faculty of Medicine, Skåne University Hospital, Lund University, Lund, Sweden

## Abstract

**Background:**

Severe perineal trauma sustained during childbirth is a serious complication since it can lead to both short- and long-term consequences for women. Some of the methods used to prevent perineal injuries have been evaluated in clinical trials, but there are still gaps in the evidence. A new clinical practice has been introduced, adopted by more than half of the maternity wards in Sweden with the aim of reducing severe perineal trauma. This procedure involves two midwives assisting the woman during the second stage of labour.

**Methods/design:**

In this multicentre randomised controlled trial, 2946 women will be randomised to be assisted by one or two midwives during the second stage of labour. Women age 18–47, who plan for their first vaginal birth, with a singleton pregnancy in cephalic presentation, will be asked to participate when admitted to the maternity ward. Five maternity wards comprising 19,500 births/year in different parts of Sweden will participate in this study. The sample size is powered to demonstrate a 50% reduction (from 4.1–2.0%) in primary outcome, which is the prevalence of severe perineal trauma (3rd and 4th degree). Secondary outcomes will include maternal and neonatal outcomes, women’s experiences, midwives’ experiences of the intervention, incontinence, and pelvic floor symptoms. The primary analysis is intention to treat. Questionnaires will be sent to the women at 1 month and 1 year after the birth to assess women’s experiences, pain, incontinence, pelvic floor symptoms, sexual function, and mental health.

**Discussion:**

It is important for care during labour and birth to be evidence based. There is a strong desire among midwives to reduce the risk of severe perineal trauma. This may lead to new strategies and practices being implemented into practice without scientific evidence. The intervention might have negative side effects or unintended consequences. On the other hand, there is a possibility of the intervention improving care for women.

**Trial registration {2a}:**

ClinicalTrials.gov NCT03770962. Registered on 10 December 2018

## Background {6a}

Severe perineal trauma sustained during childbirth is a serious complication since it can lead to both short- and long-term consequences for women. It is the most important cause of female anal incontinence, and the reported prevalence following severe perineal trauma is 10–30% [1, 2]. Other complications of severe perineal trauma are pain [3], dyspareunia [4], an altered body image, and psychosocial problems [5–7]. Risk factors for severe perineal trauma are giving birth vaginally for the first time, having an assisted vaginal birth, giving birth vaginally after a previous caesarean section, or giving birth to a baby that weighs more than 4000 g, ethnicity, and the risk increases with age [8–11]. Some of the methods used to prevent perineal injuries have been evaluated in clinical trials, but there are still gaps in the evidence [12]. With high level scientific evidence lacking for most of the preventive strategies midwives use (except for perineal warm compresses) [12], most midwives believe that a slow and controlled birth is a key factor in prevention. How this slowing of the birth should be undertaken continues to be debated [13]. Furthermore, there is a lack of knowledge about how women experience the second stage of labour, the care they receive, and their perspectives on the methods midwives use to facilitate birth and prevent perineal trauma [12].

The second stage is considered to be the most stressful part of the labour for the woman and her unborn baby, and consequently also for the midwife [14]. Traditionally, midwives in Sweden have asked other midwives for a second opinion, for assistance only in complicated situations or in obstetric emergencies. Recently, a new clinical practice has been introduced in approximately 50% of the maternity wards in Sweden to reduce severe perineal trauma [15]. This procedure involves two midwives attending the woman during the second stage of labour regardless of risk. The primary midwife who is responsible for the birth calls for the second midwife when the active phase of the second stage has started and the presenting part of the baby is visible. While this is common practice in many other developed nations, it is not in Sweden, where midwives are assisted by assistant nurses. An unpublished survey from one maternity ward in Sweden showed that most of the midwives appreciated this clinical practice but were uncertain as to whether it reduced the prevalence of severe perineal trauma [15].

## Aim {7}

The purpose of this study is to evaluate a clinical practice to reduce severe perineal trauma. This clinical practice involves collegial midwifery assistance during the second stage of labour, where an additional midwife is present during the active phase of the second stage of labour and the birth of the baby. The clinical practice will be compared to standard care in Sweden where one midwife assists the woman during the second stage and the birth of the baby.

## Methods

### Study design and setting {8}

This is an open label parallel multicentre randomised controlled trial named *One Plus One Equals Two—will that do*? The trial will be performed at two University Hospitals that have two maternity wards under each and one county hospital in Sweden, from 10 December 2018 to 10 December 2020. The maternity wards at Karolinska University Hospital Huddinge and Solna together have approximately 7700 births annually, and the Southern region maternity wards Lund and Malmö and Lund 9000 births, while Karlstad county hospital comprises 2800 births per year, covering approximately 18% of the total population. The trial is designed according to CONSORT guidelines for clinical trials [16], and the Standard Protocol Items: Recommendations for Interventional Trials checklist (Additional file 1) [17] was used when writing this report. The trial was registered in ClinicalTrials.gov on 10 December 2018, NCT03770962, and the first version of the protocol was uploaded on 3 December 2018.

### Participants and enrolment {9, 10, 26a}

Women who are eligible to participate in the trial will be between 18 and 47 years old, having a singleton baby in cephalic presentation, with a gestational week > 37 + 0, and plan for their first vaginal birth. Women will be excluded if they have a multiple pregnancy, have a planned caesarean section, or are < 37 weeks pregnant.

General information regarding the study will be found on each of the participating clinic’s website. Furthermore, oral information will be given, and information leaflets will be distributed at prenatal birth preparation classes held at each of the maternity wards. However, the prenatal classes are voluntary and do not cover all women who give birth at the different hospitals. Women who meet the inclusion criteria will be asked to participate when admitted to each of the participating maternity wards with contractions or for induction of labour. Written information and consent forms are available in Swedish, English, Arabic, and Farsi (Persian). The women who consent to participate in this trial will be sent questionnaires 1 month and 1 year following the birth.

### Intervention and standard care {6b, 11a, 11b}

The women enrolled in the trial will be randomised to either have one midwife assisting the active phase of the second stage of labour and the birth of the baby (standard care) or have two midwives present. If the woman is randomised to the intervention, the midwife responsible for the birth and assisting the woman asks a second midwife to be present in the birthing room when the active phase of the second stage has started and the presenting part of the baby is visible. The second midwife is ready to assist the primary midwife if needed. There will be occasions where the group allocation is not possible to follow, but intention-to-treat protocol will be followed. In cases of fetal distress, the primary midwife will always ask a senior midwife for assistance even if the woman is allocated to having one midwife. Another possible situation that could occur is where there is an extreme workload due to a busy shift and there is no possibility of accessing a second midwife. The midwives who record the data in the clinical registration forms will report all the situations where the allocated group is not possible to follow.

### Randomisation {16a, 16b, 16c, 17a}

Women who have consented to participate in the trial will be randomised to having either one or two midwives present during the active phase of second stage of labour. The randomisation will take place after the woman has entered the second stage and is performed on a 1:1 basis. Treatment group is allocated using sealed opaque envelopes. The envelopes will be prepared with a unique code consisting of a first code letter identifying each study site followed by a consecutive number. Research midwives responsible for the trials at each study site will provide the randomisation using a computer-based randomisation program, envelopes, and code lists. Code lists will be stored and kept safe at Region South Lund. The participating maternity wards are organised slightly different, but all of them have a senior midwife who is in charge and organises the care at each work shift. The envelopes are placed in a holder at a work desk in the centre of the maternity ward where the senior midwives have their workplace. The midwife who is attending the woman in labour draws an envelope together with the senior midwife in charge. It is not possible to blind the women or the midwives participating in the study due to the nature of the intervention. The data analyst from Forum South who performs the analyses will be blinded to the group allocation for the intention-to-treat analysis.

### Primary and secondary outcomes {12}

Primary outcome measure is the difference between the two treatment arms in the proportion of participants with severe perineal tears, i.e. third-degree and fourth-degree perineal tears engaging the external and/or internal anal sphincter muscle, anal epithelium, or rectum (ICD-codes O70.2 or O70.3).

For the secondary short-term outcome measures, the difference will be calculated between the two treatment arms in the proportion of participants with second-degree tears, deeper vaginal tears, first-degree tears, intact perineum (no tear), labial and periurethral tears, episiotomy, postpartum bleeding > 500 ml, birth position, and instrumental delivery, and the proportion of women who breastfeed within 2 h after the birth. Secondary outcomes regarding the newborn baby are the difference between the two treatment arms in the proportion of newborns with an Apgar score < 4 at 5 min, umbilical cord blood gases (arterial < 7.05, venous < 7.17), and admission to the neonatal intensive care unit (NICU).

Both the primary midwife responsible for the birth and the second midwife will report how they find the experience in the clinical registration forms. In addition, focus group interviews will be conducted to get a deeper understanding of how midwives experience this way of working. Women’s experiences of being attended by two midwives during the second stage of labour will be assessed via a questionnaire 1 month following birth. Women will also be asked about their experience of different methods used during the second stage to prevent perineal trauma. The questions asked are study specific, with Likert scale answers that have been tested for face-validity with 10 women who recently gave birth to their first child and then further tested in two focus groups with women. The short-term secondary outcome measures in this questionnaire will be analysed as the difference between the two treatment arms in the proportion of participants with perineal pain, use of pain medication, and self-reported mental health. Psychological well-being is measured by the Edinburgh Postnatal Depression Scale [18].

**Fig. 1:**
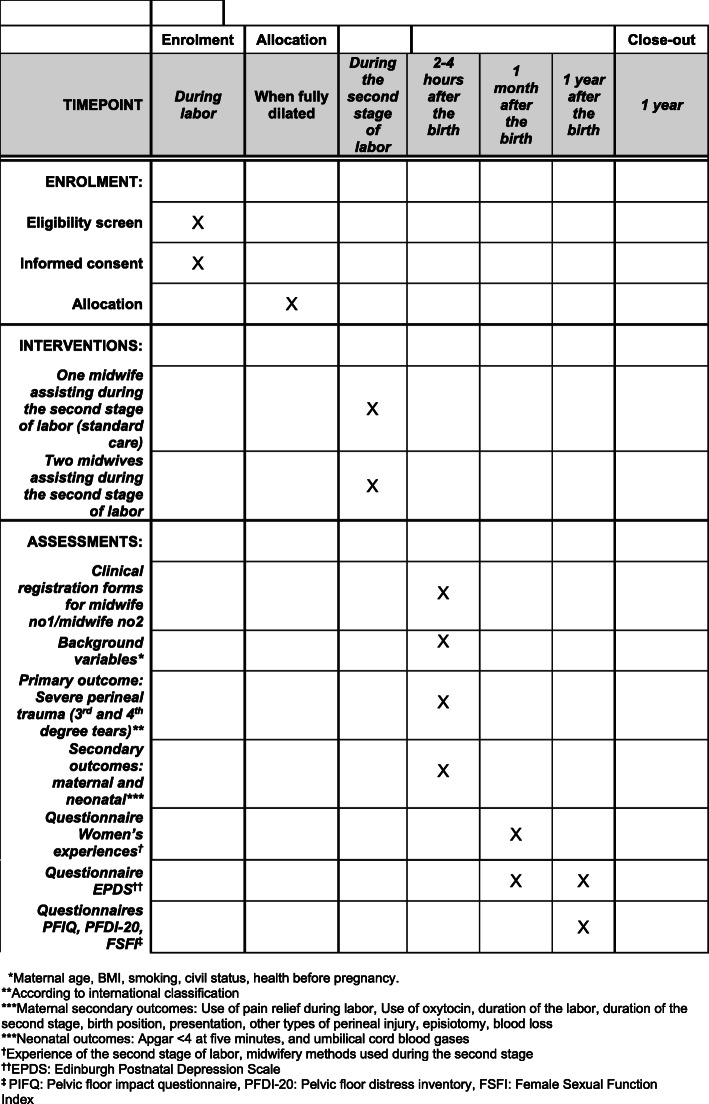
One Plus One Equals Two—will that do? Schedule of enrolment, interventions, and assessments

The long-term secondary outcomes 1 year after birth are the difference between the two treatment arms in the proportion of participants with urinary incontinence, anal incontinence, and symptoms of pelvic organ prolapse; self-reported mental health; and sexual function. For the 1-year follow-up, the following validated questionnaires will be used: Pelvic Floor Impact Questionnaire (PFIQ7, score), Pelvic Floor Distress Inventory (PFDI-20, score), the Female Sexual Function Index (FSFI, score), and Edinburgh Postnatal Depression Scale (EPDS, score) [18–20]. For EPDS, the recommended cutoff score > 12 will be used both at 1 month and 1 year after birth. The schedule of all follow-up assessments is illustrated in Fig. 1.

### Assessment and classification of perineal tears {11c, 18a}

The routine in Sweden is to offer all women who have given birth vaginally a digital rectal examination to determine the extent of the tear. After the placenta is delivered, the midwife responsible for the birth performs the initial diagnosis of the tear [21]. According to the guidelines at all the participating clinics, midwives are responsible for the first examination of tears and suturing of first- and second-degree tears. When a tear is suspected, adequate pain relief is given before the examination to enable a rectal examination. If severe perineal trauma is suspected, an obstetrician is contacted to further classify the tear. Complicated vaginal, second-degree tears and tears that involve the anal sphincter are sutured by obstetricians. All tears grade III and IV are sutured in the operating theatre.

To objectively validate the classification of the tears in this study, the midwife will examine the woman together with a midwife (or an obstetrician) who has not otherwise been involved in the birth. The midwife responsible for the birth will register the grade of the tear in the clinical registration form. In this form, the midwife states both the grade of the tear and the muscles and/or fascia involved. If an obstetrician sutures the tear, they will complete the questions regarding classification and suturing in the clinical registration form.

### Educational sessions for midwives {11c, 11d, 18a}

Before the start of the trial, educational sessions are held at all clinics with all midwives attending. The aim of the educational session is to ensure that all midwives are well informed regarding participation in clinical trials and the practical aspects and procedures of the *One plus One* trial as well as to promote participant retention.

The midwives receive written information and give their written consent to participate in the trial. At the maternity wards of Malmö, Lund, and Karlstad, the educational sessions have included theoretical training in anatomy, assessment, and classification of perineal tears and suturing techniques. The maternity wards of Karolinska University Hospital Solna and Huddinge participate in a county educational training programme for midwives and gynaecologists/obstetricians started in 2017 funded by the Stockholm County. This educational training programme focuses on reducing severe perineal trauma, pelvic floor anatomy, classification, and suturing of perineal trauma. All the participating maternity wards have a research midwife who is responsible for the study implementation, and there will be additional educational sessions for new midwives throughout the trial. All preventive strategies that midwives use will be assessed in the clinical registration forms completed by the midwives after each birth. Therefore, there are no other interventions which may be sought by the participants to complement the trial.

### Sample size and statistical analysis {14, 20a, 20b, 20c}

The Swedish Pregnancy Registry is a Certified National Quality Registry initiated by the Swedish Healthcare. It collects and processes information all the way from early pregnancy to a few months after birth [22]. Data from the Register show that 4.1% of first-time mothers suffered severe perineal trauma in Sweden 2017. To be able to detect a 50% reduction in severe perineal trauma grade III–IV from 4.1 to 2.0% with 80% power and a 5% significance level (alpha error), 1052 women in each group will be needed. The 50% reduction is based on what we have considered possible to achieve and clinically significant. Five of the maternity wards who report to the Swedish Pregnancy Register accomplished a rate below 2.0% in 2017 [22]. Allowing for a possible drop-out rate of 20% and another 20% who have obstetric emergencies and therefore unable to complete assigned protocol, this will result in 1473 women in each group and 2946 women in total.

Descriptive statistics will be used to present the data. The means, SD, median, quartiles, and 95% CI will be calculated when appropriate as will frequency tables, stratified by the two arms. The primary statistical analysis is intention to treat, but a per-protocol analysis will also be performed. For comparison between the two groups, *t* tests will be used for continuous variables and chi-square tests for dichotomous variables. A possible preventive effect of having two midwives attending the second stage of labour and birth of the baby will be calculated as a relative risk of severe perineal trauma (tear grade III–IV) with 95% CI. We will adjust for study site using logistic regression. If baseline variables are not balanced by randomisation, we will adjust for those using multivariate logistic regression. Response analyses regarding primary outcome will be undertaken at each study centre.

Secondary outcomes will be compared using *t* tests for continuous variables and chi-square tests for dichotomous variables. The Mann-Whitney *U* test will be used for outcomes based on scores.

If there is substantial missing data, the variable with missing data will be handled in collaboration with a statistician from Clinical Studies Sweden, Forum South. The appropriate method for handling missing values will be chosen depending on the quality of the data.

### Data collection {18a, 18b}

Data on background variables will be obtained electronically through the database at each participating clinic. Four of the maternity wards use Obstetrix© (Siemens), and one uses Cosmic© (Cambio). After each birth, the midwives will complete a clinical registration form. The clinical registration forms will be coded with the same code as the participating woman received when she was allocated to the intervention or standard care. A data program to enter the data from the clinical registration forms has been acquired, which enables a second check of values, and those who enter data will be trained.

#### Clinical registration form for the primary midwife

This form contains questions regarding the woman, labour and birth variables, methods of preventing perineal trauma, diagnosis of the tear, and how the tear was sutured. Registered third- and fourth-degree tears will be validated through data from patient records merged from each maternity ward’s local database. If a second midwife has been present during the second stage, the midwife responsible for the birth will answer questions regarding the assistance from the second midwife and how this assistance was experienced.

#### Clinical registration form for the second midwife

When the woman is randomised to two midwives, the second midwife will also complete a clinical registration form. This form contains questions on the assistance given during the second stage of labour (if any) and how she experienced being present during the second stage of labour.

For the women participating in the trial, data will be collected through questionnaires 1 month and 1 year after the birth as described previously.

### Data management {19, 27}

Signed consent forms are collected by the research midwife responsible for each study site and forwarded to Region South Lund for safe keeping. Data recorded from each birth on clinical registration forms will be entered into SPSS© (IBM SPSS Software) by the investigators and merged with background data from the local database (Obstetrix©, Cosmic©). The data will be available only to the investigators. The clinical registration forms and background data will be merged using the women’s security number. Once the data is merged and the dataset is cleaned, the women’s security number will be deleted and the code number given when randomised is used to ensure anonymity to the participants. All data will be kept locked in a fire safe cabinet at Region South Lund. The code lists that link participating women’s security number with code numbers will not be stored in the same cabinet as the clinical registration forms. Trial data will be stored for 10 years and then destroyed as per ethics requirements. Clinical Studies Sweden, Forum South, has assessed the trial protocol and given valuable feedback for improvements regarding monitoring and safety of the trial. Clinical Studies Sweden, Forum South, is a freestanding support organisation for all health care professionals regarding clinical studies.

### Data monitoring {21a, 21b, 23}

The study is not monitored by an independent monitor. The research midwives at each site will continuously discuss the study conduct process with the PI and the regarding protocol compliance. The steering committee will also handle ethical issues that might arise.

### Potential harms {22, 23}

As approximately 50% of the maternity wards in Sweden already have implemented this clinical practice without reporting any adverse side effects, such as increased rates of severe perineal trauma or an increase in Apgar scores < 4 at 5 min, there will be no data monitoring committee and no regular audits or routine interim analyses throughout the data collection. In this study, it is not applicable to collect adverse events in the normal sense, that is, to collect them systematically (ie, question specifically) or non-systematically (i.e. by spontaneous report), etc.), since the intervention is the presence of one or two midwives, and both practices are already in clinical use. Furthermore, the primary and secondary outcomes are to a great extent covering the negative events to be foreseen, which are part of the clinical routine to document in the medical record to secure patient safety for both the mother and the baby. If the maternity wards pay attention to a negative trend in their follow-ups of outcomes related to the mother (severe perineal trauma, postpartum haemorrhage > 1000 ml) or the baby (Apgar scores and neonatal resuscitation), this will be reported and discussed with the steering committee. If a negative trend is reported to the steering committee, they will decide on how to proceed and whether it is necessary to temporarily stop the trial and perform analyses. Four of the five participating maternity wards register their data in the Swedish Pregnancy Register [22] and have immediate access to these outcomes. The fifth maternity ward has access to all the data through Cosmic©.

### Patient involvement

For this trial, we have involved women in the design of the study, for the primary and secondary outcomes and the ethical considerations. However, the greatest involvement of women’s perspectives has been in the construction of the questionnaires that are sent to women at 1 month and 1 year following the birth.

## Discussion

The purpose of this study is to evaluate a midwifery intervention created to potentially prevent severe perineal trauma. The hypothesis is that an additional midwife during the active second stage of labour will reduce severe perineal trauma compared to standard care.

Severe perineal trauma is of major concern for women as it is associated with both short- and long-term quality of life consequences [6, 23, 24]. Even though the rate of severe perineal trauma has decreased in Sweden during the past years [22], there is still a lack of knowledge regarding effective preventive strategies and a need for more research in the field [12]. Currently, there is an ongoing debate in the Swedish media and among women regarding consequences of perineal injuries including severe perineal trauma and deep vaginal and second-degree tears [25]. Childbirth organisations are upset that care providers are not able to prevent injuries and find it extraordinary that gaps in health care knowledge in this field still exist [25].

As all professions involved are aware of the potential consequences for women, there is a strong desire to do everything possible to reduce the risk of severe perineal trauma [26]. The emotive nature of the trauma and a desire to prevent it may lead to new strategies and practices being implemented into practice without first being evaluated scientifically. The practice of having an additional midwife present during the active phase of the second stage of labour may be a non-invasive strategy compared to other preventive interventions [27]. However, adding another midwife in the birthing room can be viewed as a complex health care intervention [28] which may have negative side effects or unintended consequences. The maternity wards that have employed the method have not increased the numbers of midwives. It is possible that other women in labour will be left unattended when the midwives try to undertake this new practice in the clinical setting. Since midwives are used to assisting each other during births when complications occur, one might hypothesise that this clinical practice might lead to more interventions during the second stage of labour in uncomplicated births. On the other hand, there is a possibility of learning from each other and using the intervention as a way of implementing evidence-based care. How midwives share the responsibility during the second stage in the birthing room and how they communicate, and if they reflect and give each other feedback are factors that will be further evaluated in focus group interviews. While other countries have two midwives present for the birth, this has not been common practice in Sweden, and hence, this presents a unique opportunity to inform both women and practitioners in Sweden and beyond.

The strengths of this study are the randomised design and the detailed clinical registration forms completed by the midwives. Randomised controlled trials are viewed as the most reliable method of determining the effectiveness of a treatment or an intervention [29]. As several maternity wards participate in the trial, this will increase the generalisability of the results. A response analyses regarding the primary outcome will try to highlight an eventually observer effect, an aspect known to exist when the study includes individuals’ behaviour in response to the awareness of being observed.

Previous research acknowledges that both under- and overreporting of severe perineal trauma exist [30, 31]. There can be significant oedema, bruising, and bleeding which makes it difficult to identify anatomical structures as well as discomfort during examination. This can lead to a potential misclassification of tears. To ensure correct classification of tears in this study, a second assessor (midwife or obstetrician) will assess the tear together with the midwife responsible for the birth, which is shown to increase the accuracy [30, 32]. To further increase the validity of the classification, all midwives participating in the study have received training in how to assess, classify, and suture tears before the study start. Ultrasound is sometimes proposed as a way of increasing accuracy of classification of severe perineal trauma, since it increases detection of sonographic abnormalities of the external and internal sphincter [32]. However, in a recent study when endoanal ultrasound was performed immediately following birth, the detection rate of OASIS was not significantly increased compared with clinical examination alone [30].

This study will meet several identified gaps in care knowledge. The results from this study will evaluate whether a practice with an additional midwife during the active phase of the second stage of labour is effective in reducing severe perineal trauma. Furthermore, it will evaluate other midwifery care methods used during the second stage of labour, women’s experiences of these preventive methods, and women’s physical and psychological health 1 year after the birth.

### Clinical significance

The number of women seeking care for pelvic floor problems related to childbirth is increasing. Improving the health and well-being for women giving birth is important on a personal level but also for society. Women are becoming increasingly afraid of giving birth vaginally because they fear an extensive tear and the consequences such a tear might have on their life. Some women are so fearful that they request a caesarean section [33]. Hence, it is imperative to fill this gap in care knowledge [34].

The results from this study will show whether this clinical practice, which approximately 50% of all maternity wards in Sweden already have adopted, is preventive or not, or if it has any negative side effects. If the presence of a second midwife can be demonstrated to be preventive, the practice can be implemented in all the maternity wards in Sweden. Otherwise, health care resources could be used more effectively. The results from this study will also generate knowledge about women’s experiences of the midwifery care methods to prevent perineal trauma. This knowledge is currently lacking and is important as care should be both effective and of value to women.

### Trial status

Karolinska University hospital Huddinge started recruiting 10 December 2018, Region South Lund delivery ward 14 January 2019, and Region South Malmö delivery ward and Karolinska University hospital Solna 4 March 2019. Karlstad County hospital started recruiting 15 October 2019. The recruitment is expected to be completed 31 December 2020.

## Supplementary Information

**Additional file 1.** SPIRIT 2013 Checklist: Recommended items to address in a clinical trial protocol and related documents.

## Abbreviations

EPDS: Edinburgh Postnatal Depression Scale
PFIQ: Pelvic Floor Impact Questionnaire
PFDI-20: Pelvic Floor Distress Inventory
FSFI: Female Sexuality Function Index
