# Correction to: One Plus One Equals Two—will that do? A trial protocol for a Swedish multicentre randomised controlled trial to evaluate a clinical practice to reduce severe perineal trauma

**DOI:** 10.1186/s13063-020-04936-5

**Published:** 2020-12-02

**Authors:** M. Edqvist, H. G. Dahlen, C. Häggsgård, H. Tern, K. Ängeby, G. Tegerstedt, P. Teleman, G. Ajne, C. Rubertsson

**Affiliations:** 1 Department of Health Sciences, Faculty of Medicine, Lund University, Lund, Sweden; 2 Clinical Epidemiology Unit, Department of Medicine, Karolinska, Institutet, Stockholm, Sweden; 3 School of Nursing and Midwifery, Western Sydney University, Sydney, Australia; 4 Centre for Clinical Research and Education, Region Värmland, Karlstad, Sweden; 5 School of Education, Health and Social Studies, Dalarna University, Karlstad, Sweden; 6 Department of Obstetrics and Gynaecology, CLINTEC, Karolinska Institutet, Stockholm, Sweden; 7 Department of Obstetrics and Gynaecology, Faculty of Medicine, Skåne University Hospital, Lund University, Lund, Sweden

**Correction to: Trials (2020) 21:945**

**https://doi.org/10.1186/s13063-020-04837-7**

After publication of the original article [1], we were notified that a typo had been included in the title.

Originally published title:

“One Plus One Equals Two—will that do? A trial protocol for a Swedish multicentre randomised controlled trial to evaluate a clinical practice to reduce severe perineal trauma {1}”

Correct title:

“One Plus One Equals Two—will that do? A trial protocol for a Swedish multicentre randomised controlled trial to evaluate a clinical practice to reduce severe perineal trauma ".

The original article has been corrected.
